# New Qualitative Perspective in Human–Computer Interaction: Designing Mobile English for STEM

**DOI:** 10.3389/fpsyg.2022.863422

**Published:** 2022-05-03

**Authors:** Karmila Rafiqah M. Rafiq, Harwati Hashim, Melor Md Yunus

**Affiliations:** Faculty of Education, Universiti Kebangsaan Malaysia, Bangi, Malaysia

**Keywords:** English language, human-computer interaction (HCI), mobile, needs analysis, Science, Technology, Engineering, Mathematics (STEM), vocabulary

## Abstract

The emerging field of human–computer interaction (HCI) opens up more opportunities for technology integration in language learning. Technology creates more workforces in the Science, Technology, Engineering, Mathematics (STEM) field, yet the number of STEM pursuers is declining due to poor command of the English language. There is a gap in English vocabulary for STEM, which needs a novel solution from the perspective of HCI. This study aims to explore the needs of STEM learners in creating an English language competency mobile module. The methodology used is through a qualitative study. Seven STEM learners, 17 years old, participated in semi-structured interviews. The results from the interview are divided into four main themes: (1) the importance of learning English, (2) problems of learners, (3) strategies of English language learning, and (4) learners' readiness in using a mobile app. This study is significant for mobile app designers, English language teachers, and course designers as the input will provide an overview of the STEM learners' needs in the English language. Plus, designing a mobile app from the learners' perspectives gives more effectiveness to HCI, rendering success to second language acquisition. Future work can design and develop a mobile app to enhance STEM learners' English language competency based on the learners' perspectives.

## Introduction

Human-computer interaction (HCI) was pioneered by Professor Ben Shneiderman and has since evolved with the industrial revolution, providing more fresh ideas for its integration (Shneiderman et al., 2017). The notion behind HCI is to provide an effective interaction between humans and computers, which means that any computer system design should be feasible and easily accessible for users. Though HCI is more toward the computing field, education incorporates technology, too. The emerging field of HCI opens up more opportunities for technology integration in education, particularly language learning *via* various media such as mobile applications. Technology creates more workforces in the Science, Technology, Engineering, Mathematics (STEM) field. The STEM field is vast, yet the number of STEM pursuers is declining due to poor command of the English language among non-native speakers (Lacosse et al., 2020; Lee and Stephens, 2020) because of limited vocabulary in STEM (Fang and Liu, 2020). Despite learning the English language from young, learners still find it challenging to master English (Aravind and Rajasekaran, 2020).

Vocabulary acquisition is indeed crucial in ensuring the success of language learning. Despite having other skills in the English language such as grammar, reading, writing, listening, and speaking, the mastery of vocabulary is more vital. This is because, without proper vocabulary in the language, learners will be unable to use the language in terms of accuracy and fluency (Wallace and Lee, 2020; Yunus et al., 2020). A study reported that one of the factors associated with the difficulties in learning the second language is the lack of vocabulary (Misbah et al., 2017). Similarly, studies reported that one barrier to learning English includes anxiety (Ahsan et al., 2020) due to teachers' inability to scaffold language learning (Nasr et al., 2020). In traditional classroom teaching and learning, Vygotsky mentioned the vital role to scaffold learning (Vygotsky, 1997). With scaffolding, learners would learn better. However, the pandemic has reformed education to online platforms. Thus, technology, particularly a mobile application, helps (Hashim, 2018). But before creating a mobile app, it is crucial to identify the needs of learners to ensure that the mobile learning modules cater to the needs of learners (Patel et al., 2018; Rafiq et al., 2019), rendering to the importance of HCI.

Needs analysis is indeed an essential aspect of research, especially in identifying the learning problems of learners. A study toward service technicians in the pest control industry reported that the technicians need English communication skills (Hee and Zainal, 2018). This includes listening and speaking skills, rendering a better job performance. Similar findings were reported by a study toward maritime students (Aeni et al., 2018). However, a study carried out on lawyers reported that the needs in Legal English are public speaking, acquisition of vocabulary, legal writing, and phone communication skills (Sierocka et al., 2019). This is true because Legal English has different vocabularies to General English (GE). It is one of the studies which emphasized vocabulary acquisition in a particular field.

In the context of the STEM field of studies, Rossikhina et al. (2019) reported the unexpected needs analysis findings from engineering students at a Russian technological university, whereby the most crucial skill for these engineering students is reading because English is rarely used in communication and writing. However, a more recent study reported that the needs of pharmacy learners are in terms of speaking abilities (Syakur et al., 2020). Regardless, it was also mentioned that the overall competency was important, similar to what had been stated by Ye (2020). Therefore, it is vital as a guide for researchers to identify learners' targets and learning needs, specifically in different contexts.

Based on the above-mentioned discussion, it could be seen that there is a gap in the vocabulary for STEM in English because each field focuses on different vocabulary. Therefore, it needs a novel solution from the perspective of HCI. Almost everyone owns a mobile device, which means a mobile application can create supplementary lessons for STEM learners as a new advanced platform for learning (Klimova and Pikhart, 2021; Rafiq et al., 2021). Hence, this study aims to explore the needs of STEM learners in creating the English language competency mobile module.

## Methods

### Study Design

The qualitative research method is used to explore the needs of STEM learners in creating the English language competency mobile module. The themes are identified based on the importance, problems, strategies, and mobile readiness of learners in learning English. A semi-structured interview was carried out to identify learners' needs to cater to our research aim. The interview protocol was adapted from Dudley-Evans and St John's Needs Analysis Model (Dudley-Evans and St John, 1998).

### Study Setting

Due to the pandemic, there was a restriction movement order in Malaysia. Hence, it was not possible to carry out a face-to-face interview. Dodds and Hess (2021) mentioned that there is not much difference between online and face-to-face interviews in qualitative research as long as the ethical procedures are fulfilled. Thus, we interviewed *via* Google Meet as the platform. The learners are from one public school in Malaysia. They were chosen because the school conducts dual-language STEM subjects, giving more justifications and insights regarding the learners' perspectives on learning English for STEM.

### Participant Recruitment

First, the purpose of this study was explained to one STEM class in a public school. Out of 21 students from the class, only seven volunteered to participate in the interview. The eligibility criteria include: 17 years old and must take the STEM stream in high school. Our study focuses on learners' needs in learning English for STEM *via* a mobile app. The participants were chosen *via* purposive sampling to ensure that rich data from selected participants could be obtained (Islam and Aldaihani, 2021). Participants did not receive any incentive by participating in this interview.

### Participant Consent and Data Collection Procedures

Before the interview, participants consented to use English as much as possible to answer the interview questions. They also consented to have their interview session audio-recorded. Data were collected based on semi-structured interviews, which lasted for 30 min on average. After the interview, the interview transcript was returned to respective participants for member checking to ensure the credibility and trustworthiness of the data collected. The interview constructs include four aspects: (1) importance of English, (2) problems in learning English, (3) learning strategies used in learning English, and (4) mobile readiness in learning English. Only one researcher took part in this interview session to ensure that learners did not feel pressured by the presence of many researchers. The researcher also took field notes to note on participants' non-verbal behavior. Researchers did not use any template for the field notes.

### Data Analysis for Qualitative Data

Data from the interview transcripts were analyzed using thematic analysis. The transcripts were analyzed using the Atlas.ti software. The process included six steps by Braun and Clarke (2006). The process include: (1) transcripts were read to familiarize with the data, (2) preliminary codes were assigned to describe the content of the transcripts, (3) themes in the codes were constructed, (4) themes were reviewed, (5) themes were defined, and (6) themes were named before writing a report. The researcher followed the six steps as mentioned in this study.

## Results

From the analysis, eleven subthemes emerged, categorized into four main themes: (1) Importance of English, (2) Problems in learning English, (3) Strategies in learning English, and (4) Mobile readiness in learning English. Table 1 summarizes the main themes and subthemes for this study.

**Table 1:** Summary of the main themes and subthemes.

**Themes**	**Subthemes**
1. Importance of English	1st subtheme: English is important for education 2nd subtheme: English is important for communication purposes 3rd subtheme: English is important because it is an international language
2. Problems in learning English	4th subtheme: Language related problems 5th subtheme: Self-related problems
3. Strategies in learning English	6th subtheme: Learning via texts 7th subtheme: Learning through audio-visual materials 8th subtheme: Learning through games 9th subtheme: Learning with a teacher's guidance
4. Mobile readiness in learning English	10th subtheme: Attitude of learners 11th subtheme: Learning activities

### Theme 1: Importance of English

Question: *Why do you think English is important for you as a secondary school student?*

#### 1st Subtheme: English Is Important for Education

The researchers asked participants why English is essential to identify the learners' view of learning English. Across the responses, education is the most crucial reason for learning English. English is used in many subjects of education, especially Science and Mathematics. Also, in tertiary education, most STEM courses are taught in English, making the participants aware of the importance of English. The response from Participant 6 mentioned, “*in university, everything is in English.”* Participant 1 mentioned, “*for me, learning English is easy if we start from young or maybe we keep learning and learning, like that.”* These perceptions showed that the participants had been exposed to learning English since they were young, perceiving that English is important for education.

#### 2nd Subtheme: English Is Important for Communication Purposes

Based on the responses, a few participants mentioned that learning English for communication is essential, which many learners value. Five participants said that English is vital for communication. When prompted to, one participant mentioned*, “we can talk with our idols” (Participant 5)*. Another response mentioned, “*easy to communicate with other people, can speak easily” (Participant 4)*. These responses referred to the participants' perception of learning English to communicate with other countries. One participant said, “*we will not be easily deceived by foreign people” (Participant 5)*. These responses showed that learners in high school might have the thought of going abroad; hence learning English seemed to be important to them for communication.

#### 3rd Subtheme: English Is Important Because It Is an International Language

Another importance of learning English is because English is an international language. One of the participants mentioned, “*English is the language people always use. It's wide and wherever we go, other than our own language, Bahasa Melayu, we have to have side language. The most common is English” (Participant 1)*. In enhancement, Participant 2 also quoted the word lingua franca in his response, “*The English language is one of the lingua franca languages, and almost all country in the world know English as a communicating language… Even though English is not our mother tongue language, but we have to learn it because English is also one of the lingua franca languages” (Participant 2)*. Additionally, Participant 1 added, “*and when we go anywhere, it's not only our language, but we have to learn English so that it's easy”*. Looking into these responses, the notion that English is an international language becomes one reason for learning English.

### Theme 2: Problems in Learning English

Question: *What are the difficulties or challenges that you've faced in learning English?*

#### 4th Subtheme: Language Related Problems

The researcher then asked the second question to know the learners' problems in learning English. One of the problems was related to language difficulties. One response highlighted, “*they are weak in vocab and grammar. Vocab is important because we want to write essays and all that, we use vocabs” (Participant 7)*. Another response noted*, “the problem of grammar, grammar. Lack of vocabulary” (Participant 3)*. Most of the responses revolved around grammar and vocabulary. The researcher followed up with prompt questions to know why they said grammar and vocabulary are difficult, and one response mentioned, “*because if we don't understand the vocab, we won't understand” (Participant 1)*. Language related problems that learners face are related to grammar and vocabulary. They know that they would not acquire other language skills without vocabulary.

#### 5th Subtheme: Self-Related Problems

The next problem related to English language learning is the learners themselves. The barrier is often associated with learners' shyness and lack of confidence in using English, despite having learnt it since young. Also, they are afraid of being laughed at; hence they refrain from using English. One participant mentioned, “*sometimes when they try to speak English, people will make fun of them, like judgmental society” (Participant 6)*. Another response highlighted that “*didn't get support from friends, maybe family” (Participant 4)*. These participants felt anxious when they did not have the support from their surroundings to encourage them in using English. Another response gave an example of anxiety that he felt when he spoke English, “*but for speaking, you need to have courage. It must come from your own self. No one can help in courage own effort. In speaking, especially in presentation, it is difficult to improve in a group speaking. Because sometimes, someone will speak more and some will not speak at all. It's not about group. Also, to build confidence, we present alone, but only four or five people watch. It's not like we present many members and many people watch also, the courage is difficult to build” (Participant 7)*.

### Theme 3: Strategies in Learning English

Question: *How do you learn English? What are the strategies that you use to learn English?*

#### 6th Subtheme: Learning *via* Texts

Third, the researcher asked what strategies the participants used in learning English. Though learning *via* texts emerged as the subthemes, only two participants mentioned it. Regardless of that, the researcher believed it is an exciting finding that should not be excluded from the report. One response mentioned, “*use Thesaurus” (Participant 4)*. Hence, the interviewer probed the answer:

*Interviewer: Could you explain how you use Thesaurus to improve your English?*

*Participant 4: I find similar words. [long pause]*

*Interviewer: Could you elaborate? Why do you use Thesaurus instead of Google?*

*Participant 4: Google sometimes not good. I have it at home, the Thesaurus. Thesaurus has like synonyms and antonyms and got sample sentences*.

Another participant expressed, “*Reading novels, I like them. Sometimes I read Webtoons. My favorite” (Participant 5)*. Webtoon is an online platform for comics, mainly from Korean authors, but the comics are officially translated to English. Though participant 4 preferred to use the hard copy version of Thesaurus, Participant 5 mentioned that she even read novels online because it's convenient.

#### 7th Subtheme: Learning Through Audio–Visual Materials

Audio–visual materials like videos encourage learners to learn English. Many participants mentioned they watched videos on YouTube and other platforms in the English language. One interesting response said, “*Although we [referring to him and his family] watch English movies, subtitles also English” (Participant 6)*. Generally, participants learned English by watching and learning through the subtitles. Still, one response mentioned, “*I always watch movies and anime by reading the subtitles, and if I don't understand it, I would gladly Google translate the word” (Participant 2)*.

#### 8th Subtheme: Learning Through Games

Another way of learning English, which various participants mentioned, was through games. One response said*, “from games also we can know more words like vocabulary game” (Participant 4)*. Another response also mentioned a similar answer, “*I use game word search also. I get to know more words” (Participant 6)*. On another note, one response elaborated using video games instead of word games, “*and I used to expose to English at a young age since my nephew and uncle play video games so much, I started to play it and started to understand English a little bit” (Participant 2)*.

#### 9th Subtheme: Learning With a Teacher's Guidance

Though participants agreed that they learned *via* materials, one participant specifically mentioned that learning English in class with a teacher's guidance helped him improve. He noted, “*Other than that, the learning process in class is also important to make sure our grammar, pronounce and literature good and nice. If somehow students out there didn't seem like to give a full attention on classes, maybe they won't be able to improve their English” (Participant 2)*. Though only one participant mentioned this, it is worth noting as learning English with proper guidance helps.

### Theme 4: Mobile Readiness in Learning English

Question: *How ready are you in mobile learning? What are the contents you require in a mobile application?*

#### 10th Subtheme: Attitude of Learners

Finally, the researcher asked participants' readiness in using a mobile app to learn English. Participants are positive toward using the mobile app. All the participants are ready to use a mobile app as a supplementary lesson to improve their English language competency. Some of the reasons are the mobility feature, where learners can learn anywhere, which is easy. One response mentioned, “*of course. Now no one wants to hold a heavy dictionary” (Participant 6)*. Aside from the easiness to use a mobile app, Participant 2 mentioned that people are more attached to gadgets in the current days, which will be beneficial if an app for English is created. He specifically said, “*yes, because people nowadays are more attached to mobile phones, laptops, tablets, and PC more than they attached to books. So, it's really helpful for them to have a good app to improve their English” (Participant 2)*. Additionally, Participant 1 personally mentioned the current pandemic situation, which makes learning through an app more beneficial through a mobile app. She stated that “*yes, of course. Because now during pandemic, students do nothing at home. So, for those who want to learn English, think English is difficult and want to improve their English, they can use the English apps” (Participant 1)*.

#### 11th Subtheme: Learning Activities

Learning activities are central to improving learners' English language competency through the mobile app. One participant mentioned, “*it's important to put media like audios, videos, graphics, and animations” (Participant 5)*. Another response also said that using audio–visual materials, “*video can, but the important thing is not too long, like a minute okay maybe”(Participant 5)*. Plus, one participant said, “*if we watch video, we can improve vocab” (Participant 7)*. Aside from audio–visual materials, many participants also mentioned games like quizzes and mini-games. Participants 3 and 7 noted that they are agreed on integrating quizzes in the app.

Participants 1 and 2 mentioned including mini-games to create a more engaging app. The suggestion from Participant 1 is unique as, she said, “*for example, if we play the tap app, understand? When you want to go to the next question or anything next, they'll ask you to tap this and that. Ah, that sounds like fun because it makes us feel like we want to know more, want to know more, like that” (Participant 1)*. Additionally, Participant 2 also mentioned the fun aspects of a mobile app with mini-games, “*…maybe mini-games, so that the app will not be very boring for everyone to use. A little fun would be okay” (Participant 2)*.

## Discussion

This study aimed to explore the needs of STEM learners in creating the English language competency mobile module, adhering to HCI. The freedom to learn STEM subjects in different languages might cause difficulties for learners in the future (Ye, 2020). With reference to the findings from the needs analysis, it is vital to have a well-built application, ensuring users' comfort in interacting with the system to learn the language effectively (Shneiderman et al., 2017). As highlighted in the findings, vocabulary acquisition is fundamental to language learning. This contradicts previous studies that mentioned that the essential skill in language learning is speaking skills (Aeni et al., 2018; Hee and Zainal, 2018; Syakur et al., 2020). One reason is that second language learners face challenges acquiring vocabulary for the second language; hence, they require adequate vocabulary to master all other language skills. As supported by Sierocka et al. (2019), vocabulary acquisition is crucial for different learners because the terms and jargon differ for each field of study, rendering to the importance of acquiring specific vocabulary for specific contexts.

Additionally, this study showed that many STEM learners mentioned being anxious and shy to speak English. Supporting the findings, a study that looked into the factors affecting English language learners' shyness reported that they had limited knowledge of the language, resulting in anxiety (Ahsan et al., 2020). This is an issue among second or foreign language learners, where they need to acquire vocabulary to use the English language proficiently. Without the basic knowledge of vocabulary or words in English, learners will face difficulties (Misbah et al., 2017), especially STEM graduates who need English to perform efficiently. Therefore, findings from this study could contribute to a well-designed mobile app. With a proper design of the mobile app, adhering to HCI aspects, learners will be able to acquire vocabulary any time anywhere.

Human–computer interaction prioritizes multiple interaction patterns and the openness of the design space. Participants in this study mentioned the use of audio–visual materials. However, one participant also specified the teacher's involvement. Although the focus is on technology in HCI, there is a need to look into human interaction. Therefore, findings from this study clearly showed the importance of HCI to ensure that teachers' presence is also an aspect to be considered when developing an app. In enhancement, app development *via* HCI could embed the social constructivism theory by Vygotsky (1997) to include a teacher's role to scaffold language learning. Regardless, technological elements, such as audio–visual materials are preferred by learners as supported by other studies as well (Hashim, 2018; Rafiq et al., 2021). However, media or tools should not replace teachers' role as teachers who scaffold learning will give a better outcome for learners (Nasr et al., 2020). This provides more opportunities for researchers to implement HCI constructs in designing a mobile application to enhance second language learning.

## Limitations

This study was carried out to explore the needs of STEM learners in creating an English language competency mobile module, which looked into specific samples with specific backgrounds. There are limitations to our study. First, there is a limitation in transferability. Also, the number of participants who volunteered was from one class in a public school; hence the sample might not represent the whole population of STEM learners. Despite the limitations, this study has strengths. First, this study is one of the earliest studies to look into the needs of STEM learners in second language learning from the HCI perspective. Second, this study provides evidence that learners have their preferences in learning *via* a mobile app, which can be considered when designing and developing feasible mobile applications.

## Contributions

One major significance of this study contributes to the language policy aspect. Many non-native countries teach English as either a second or foreign language, especially in higher education, because English is an international language and learning English is necessary for education, especially for learners to further their education (Wallace and Lee, 2020). With that in mind, policymakers could look into introducing English for STEM in high schools to further assist and prepare learners for higher education. Findings from this study are one of the earliest preliminary studies looking at English language, STEM and mobile integration, a multidisciplinary study. Contributing to the analysis stage in design and development, this study enlightens course developers, assisting them in developing a mobile app from the perspective of HCI. This study advances a novel perspective view for second language learning with appropriate technology design and integration *via* HCI.

## Conclusions and Implications

This study proposed exploring the needs of STEM learners in an English language competency mobile module. The results from the interview implied that STEM and English language rely on each other because language ability is an important aspect. This study also implied the importance of introducing vocabulary to second language learners first before teaching other language skills. This is because vocabulary is the foundation of language learning. Furthermore, findings from this study implied that further research on HCI to assist vocabulary learning is feasible, opening up more opportunities for future research on HCI.

This study is significant for mobile app designers, English language teachers and course designers as the findings provide an overview of the STEM learners' needs in the English language. This study looks into learners' perspectives in mobile learning, which gives more effectiveness to HCI, rendering success to second language acquisition. One notable finding from this study, which is the mobile readiness of learners, provides insight into future mobile learning research. This study is only a preliminary study, and future work could look into the HCI aspect in designing and developing a mobile app to enhance STEM learners' English language competency.

## Data Availability Statement

The original contributions presented in the study are included in the article/supplementary material, further inquiries can be directed to the corresponding author.

## Ethics Statement

Ethical review and approval was not required for the study on human participants in accordance with the local legislation and institutional requirements. Written informed consent from the patients/ participants or patients/participants legal guardian/next of kin was not required to participate in this study in accordance with the national legislation and the institutional requirements.

## Author Contributions

KRMR, HH, and MMY conceived the study, participated in its design and coordination, and performed final analyses and co-drafted the manuscript. KRMR collected field data, entered study data, assisted in data analysis, and interpretation of study results. All authors read, revised, and approved the final manuscript.

## Funding

Universiti Kebangsaan Malaysia funded this research with Grant No. GG-2021-003.

## Conflict of Interest

The authors declare that the research was conducted in the absence of any commercial or financial relationships that could be construed as a potential conflict of interest.

## Publisher's Note

All claims expressed in this article are solely those of the authors and do not necessarily represent those of their affiliated organizations, or those of the publisher, the editors and the reviewers. Any product that may be evaluated in this article, or claim that may be made by its manufacturer, is not guaranteed or endorsed by the publisher.
